# Hazardous Interactions Between Food, Herbs, and Drugs in the First Stage of Biotransformation: Case Reports of Adverse Drug Interactions in Humans

**DOI:** 10.3390/ijms26115188

**Published:** 2025-05-28

**Authors:** Bożena Bukowska, Anna Grzegorowska, Eliza Szczerkowska-Majchrzak, Karol Bukowski, Kornelia Kadac-Czapska, Małgorzata Grembecka, Marlena Broncel

**Affiliations:** 1 Department of Biophysics of Environmental Pollution, Faculty of Biology and Environmental Protection, University of Lodz, ul. Pomorska 141/143, 90-236 Lodz, Poland; anna.grzegorowska@edu.uni.lodz.pl; 2 Department of Ecology and Vertebrate Zoology, Faculty of Biology and Environmental Protection, University of Lodz, ul. Banacha 12/16, 90-237 Lodz, Poland; eliza.szczerkowska@biol.uni.lodz.pl; 3 Department of Medical Biophysics, Faculty of Biology and Environmental Protection, University of Lodz, ul. Pomorska 141/143, 90-236 Lodz, Poland; karol.bukowski@biol.uni.lodz.pl; 4 Department of Bromatology, Faculty of Pharmacy, Medical University of Gdansk, 107 Al. Gen. J. Hallera, 80-416 Gdansk, Poland; kornelia.kadac@gumed.edu.pl (K.K.-C.); malgorzata.grembecka@gumed.edu.pl (M.G.); 5 Department of Internal Diseases and Clinical Pharmacology, Medical University of Lodz, ul. Kniaziewicza 1/5, 91-347 Lodz, Poland; marlena.broncel@umed.lodz.pl

**Keywords:** cytochrome P450, food–drug interactions, grapefruit, herb–drug interactions, monoamine oxidase, tyramine

## Abstract

Food components and herbal substances can inhibit or enhance the therapeutic effects of drugs, thus influencing their efficacy and safety. As relatively little in known of these interactions, the aim of this review is to shed further light on the potentially dangerous influences that food and herbs may have on cytochrome P450 enzyme (CYP) and monoamine oxidase (MAO) activity in the first stage of drug biotransformation. The review includes documented cases in which such interactions have led to health complications in patients. For example, fruit juices, such as grapefruit juice, cranberry juice, and pomegranate juice, have been found to interact with drugs, and to particularly inhibit CYP450 activity, and commonly used herbs are known to inhibit (e.g., *Astragalus membranous*) or induce (e.g., *Hypericum perforatum*) CYP enzymes involved in drug metabolism. CYP is also induced by polycyclic aromatic hydrocarbons (PAHs), found in grilled meat and tobacco smoke. The paper also discusses the toxic effects of tyramine, present in *inter alia* blue cheese, resulting from interactions with MAO-metabolised drugs. Most importantly, while the quantity of food and herbs consumed plays a significant role in the described drug interactions, it is possible for toxic effects to be observed even after the consumption of relatively small amounts. Patients are encouraged to consult a healthcare provider about any potential drug interactions that may occur when starting a new medication.

## 1. Introduction

Recently, considerable growth in the sales of pharmaceuticals [1] and dietary supplements [2] has been observed, and this has driven more intensive research into the potential interactions between these xenobiotics and the foods we consume. The Food and Drug Administration (FDA) describe food–drug interactions (FDIs) as changes in the pharmacokinetic or pharmacodynamic properties of a drug or nutrient, or as a decline in nutritional status caused by the introduction of a pharmaceutical agent [3,4]. Such effects can also occur as a result of interactions between the drug and the components of herbs, known as herb–drug interactions (HDIs).

These interactions have clinical significance, as they decrease or enhance the therapeutic effect of the drug, thus influencing both its efficacy and safety. Lacruz-Pleguezuelos et al. described the FooDrugs database, which contains a total of 3,430,062 potential FDIs [5]. However, despite numerous publications and case reports detailing adverse drug interactions, as well as various databases, knowledge of this area among physicians and patients remains limited [6,7,8].

Food and herbal substances can interact with the enzymes and transporters involved in drug metabolism, which may alter the concentrations of the drug in the blood. Such changes could directly affect the safety and effectiveness of the treatment [9].

During the biotransformation of xenobiotics, intestinal and liver enzymes from the cytochrome P450 family play the most important role [10,11]. A similar role is also played by monoamine oxidase (MAO); however, while CYP450 is responsible for the metabolism of 73% of drugs, MAO metabolises approximately only 1% [12,13]. The influence of food and herb compounds on drugs in humans at the biotransformation stage is a little-studied area in toxicology. Despite the existence of many in vitro or in vivo animal studies [14,15] showing these interactions, only a small number of cases have been described in humans [16,17,18].

Given the significant influence of the chemical compounds present in food and herbs on drugs, the aim of this article is to describe the types of interactions that occur during the initial stage of drug biotransformation, with a particular focus on cases reported in humans and documented in the literature. To achieve this, this study presents a number of cases concerning drug–food and drug–herb interactions occurring at stage I of biotransformation.

Case descriptions offer a number of advantages that enable healthcare professionals, such as physicians and pharmacists, to better recognise the potential risks of FDIs and HDIs, which may be rare and not obvious. Hence, all cases included in the present article document real-world clinical situations that may not yet be described in the scientific literature or textbooks; as such, our research provides valuable insights into this area, enabling the identification of new, previously unknown interactions. In addition, case descriptions can serve a didactic function, by providing examples for medical, pharmaceutical, and dietetic students and supporting the professional development of medical personnel, sensitising them to the issue of drug interactions with food and herbal preparations. Furthermore, analysing specific cases can provide a better understanding of the associated risk factors and enable the implementation of more effective poisoning prevention strategies, such as warnings on drug packaging or public education.

However, although case reports on FDIs and HDIs can be valuable for generating research hypotheses and illustrating unique clinical scenarios, their overall scientific and clinical utility is limited. They are often subject to small sample sizes, lack of generalizability, potential publication biases, and an inability to establish definitive causal relationships. These issues are compounded by confounding factors such as polypharmacy, incomplete clinical data, and interindividual variability arising from genetic and metabolic differences.

The literature included in this review was selected and evaluated systematically. The majority of articles were published between 2014 and 2024. Briefly, a search was performed of relevant original research articles and review papers in the PubMed, MDPI, Frontiers, Elsevier, and Springer databases, in addition to Google Scholar. The search was performed using the following keywords: “Cytochrome P450 and interactions”, Cheese effect, Food–drug interactions; “Grapefruit and interactions”; “Herb–drug interactions”, Monoamine oxidase, Tyramine, “Seville orange and interactions”, “St. John’s wort and interactions”, “Pomegranate and interactions”, “Pomelo and interactions”, “*Punica granatum* and interactions”. In total, the search identified 217 papers that were considered to be relevant to the topic of this review.

## 2. The Fate of Xenobiotics in the Body

**Figure 1:**
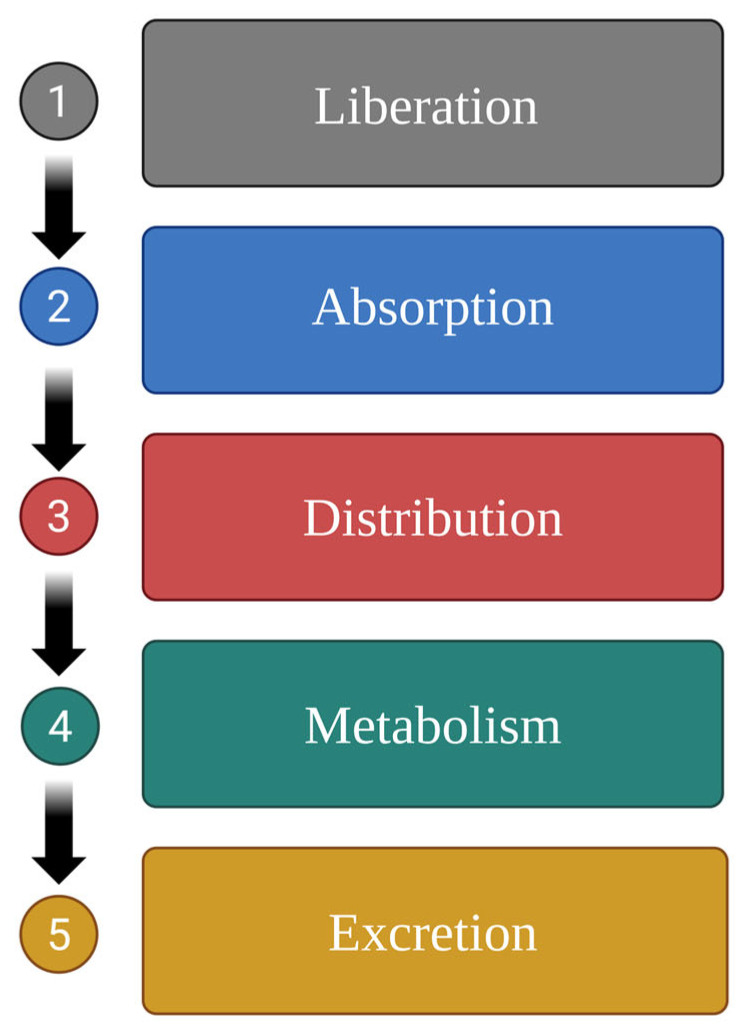
Process of drug excretion from the body following ingestion; the five stages of drug pharmacokinetics [19]. Created in BioRender.com, Agreement number QE28BF46GM dated 27 May 2025.

Pharmacokinetically, the fate of xenobiotics in the body involves the following five stages: drug liberation, absorption, distribution, metabolism (biotransformation), and excretion (LADME) [19]. Each of these stages involves distinct biochemical reactions. The primary objective of these processes is to assimilate and eliminate the exogenous substance from the body (Figure 1).

Food and herb compounds can interact with the drugs at various stages as follows [8,20]:

I. At the stage of drug release, the active substance is released from the pharmaceutical form (e.g., tablets and capsules) and becomes available for absorption in the body. During this process, the drug can bind to food components, which may hinder its release or absorption; also, the stomach pH can influence its solubility and subsequent release. Other important factors include delayed gastric emptying and interactions with digestive enzymes [21].
II. During the absorption stage, the bioactivity and bioavailability of the drug are altered, and its concentration changes depending on the type of food consumed. Absorption is influenced by changes in pH [22,23], drug adsorption, complexation, and precipitation. Furthermore, food can alter the rate of bile acid secretion, intestinal metabolism, transport kinetics, gastric emptying time, and drug properties (e.g., solubility, logP, and ionisation) [24].
III. In the distribution stage, the mechanisms that govern the distribution of the substance are also disrupted. Once absorbed from the site of administration, the drug is distributed into extracellular fluids, where it can accumulate in substantial reserves by binding to plasma proteins; this reservoir can lead to prolonged effects by establishing a sustained release mechanism [25]. In addition to various food compounds, such as cholesterol, which affect *inter alia* transport proteins, drug distribution is also influenced by the action of drug transporters, particularly P-glycoprotein (Pgp); this plays a significant role in drug absorption in the intestine, its distribution to the brain, lymphocytes and placenta, as well as excretion in urine and bile. In the intestines, Pgp reduces the absorption of toxic compounds from food, while in the liver and kidneys, it mediates the excretion of toxins and metabolites into urine and bile. Therefore, the inhibition of Pgp by food and herbal compounds in the intestines can lead to increased drug bioavailability, while its induction reduces bioavailability [26,27].
IV. During the metabolism (biotransformation) stage, the activity of enzymes involved in the metabolism of drugs or food and herbal components may be impaired or enhanced.
V. In the excretion stage, both xenobiotics and food can hinder the elimination of specific compounds. For example, a diet that acidifies urine (e.g., meat, fish, eggs, and cheese) can reduce the excretion of salicylates, sulphonamides, and ampicillin, while one that alkalinises urine (e.g., milk, vegetables) can reduce the excretion of amphetamines, theophylline, and erythromycin [28].

Of these stages, stage III is probably the most significant with regard to FDI and HDI, as it involves biotransformation, which can have dangerous effects on the health and life of patients.

### Biotransformation of Xenobiotics

**Figure 2:**
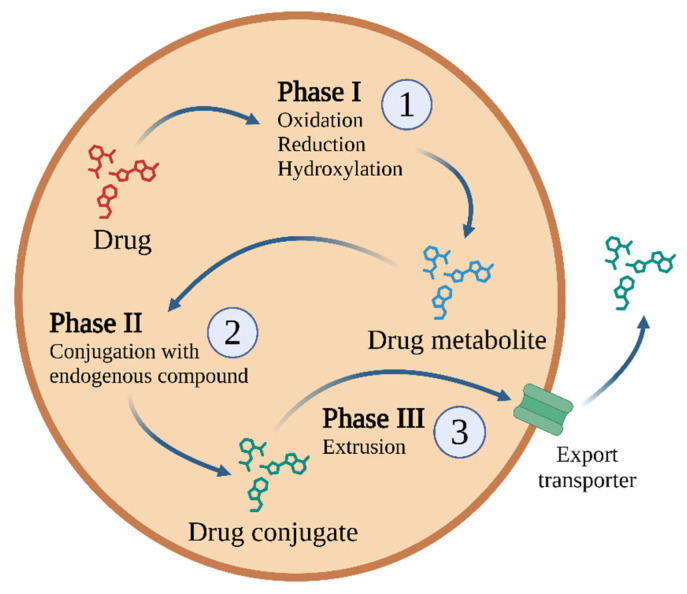
One possible biotransformation scheme used for most drugs and other xenobiotics, comprising three phases of detoxification [29]. Other drugs may not be metabolised, or may not generate conjugated metabolites (e.g., metformin [30] or gabapentin [31]). Created in BioRender.com. Agreement number IQ28BF5TV8 dated 27 May 2025.

Biotransformation is responsible for the biochemical transformation of xenobiotics to increase their hydrophilicity and facilitate their excretion. The goal of biotransformation is to convert xenobiotics into less toxic, water-soluble polar compounds. These transformations occur in three phases, with specific enzymes and transporters involved in each phase (Figure 2).

**Figure 3:**
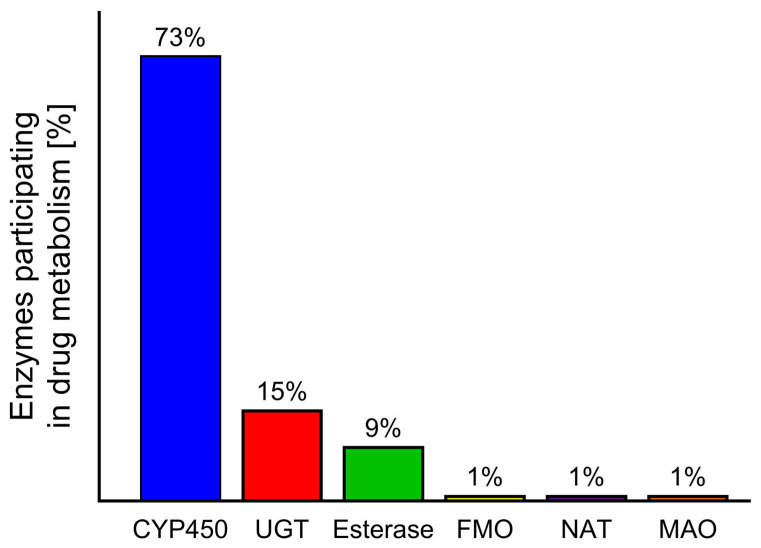
Contribution of different enzymes to drug metabolism: cytochrome P450 (CYP450), UDG glucuronosyl transferase (UGT), flavin-containing monooxygenase (FMO), N-acetyltransferase (NAT), monoamine oxidase (MAO) [11,34].

Phase I enzymes catalyse the oxidation, reduction, or hydrolysis of mainly lipophilic xenobiotics to more polar molecules [32]. The most important of these is the CYP450 superfamily, but significant roles are also played by flavin-containing monooxygenases (FMOs) and NAD(P)H oxidoreductases—quinone (NQO), amine oxidases, alcohol dehydrogenases, esterases, and peroxidases [33] (Figure 3).

CYPs are a family of haemoproteins that contain iron atoms in their structure. They bind to membranes and play key roles in the detoxification of xenobiotics, as well as in cell metabolism and maintenance of homeostasis. In addition to their primary role in drug elimination, CYPs also affect the reactions and effects of drugs, as well as their safety and bioavailability; they also partially determine drug resistance by their metabolism [11].

Cytochrome families and subfamilies are divided based on their spatial structure. There are three CYP P450 isoenzymes as follows: CYP 1, CYP 2, and CYP 3. They have low substrate specificity, making it possible to metabolise a wide range of xenobiotics. The genetic basis of the CYP450 group consists of 57 CYP genes, which are divided into 18 families and 44 subfamilies, encoding more than 50 isoenzymes [35]. CYP450 are found throughout the body, but their highest activity is observed in cells of the liver [36] and the small intestine [37].

The CYP450 protein is part of the monooxygenase enzyme family, which consists of the following three main components: cytochrome P450 (a haemoprotein), flavoproteins (such as cytochrome P450 reductases), and phospholipids. The flavoprotein, also known as cytochrome P450 NADPH reductase, contains the following two prosthetic groups: FAD (flavin adenine dinucleotide) and FMN (flavin adenine mononucleotide). These prosthetic groups facilitate the transfer of electrons to cytochrome P450. Phospholipids are crucial for the proper fusion of cytochrome P450 with flavoprotein [38]. Monooxygenases catalyse the breakdown of various substances through a hydroxylation reaction that involves the electron donor NADPH or NADH and molecular oxygen [39]. The monooxygenation reaction follows the following scheme:

RH + O_2_ + NADPH + H^+^ → ROH + H_2_O + NADP^+^

Many therapeutic drugs undergo intestinal [40] and liver metabolism [41]. The liver alone accounts for the biotransformation of approximately 75% of ingested drugs [42], while the intestinal tract plays an important role in first-pass metabolism.

Pharmacokinetic research often focusses on the relationship between the test drug and other compounds that interact with drug-metabolising enzymes. The oxidative biotransformation of most commercially available drugs is carried out by CYPs. Of the 57 functional human CYP450s, the predominant forms expressed in the liver are CYP1A2, CYP2B6, CYP2C8, CYP2C9, CYP2C19, CYP2D6, and CYP3A4/5; these are often involved in both drug metabolism and FDI/HDI processes [41]. In contrast, in the intestinal tract, CYP3A demonstrated the highest activity, followed by 2C9 (15%), 2C19, 2J2, and 2D6; however, as demonstrated in the liver, considerable variability is noted between individuals regarding the expression of individual P450 enzymes [43].

During detoxification, the xenobiotic is supplemented with polar groups by Phase I reactions. These serve as functional sites that allow for subsequent conjugation reactions catalysed by Phase II enzymes [32] such as glutathione S-transferases (GST), N-acetyltransferases (NATs), sulphotransferases, and UDP-glucuronosyltransferases. Such reactions include glucuronidation, glutathione conjugation, methylation, acetylation, sulphation, and amino acid conjugation. In all cases, the endogenous hydrophilic group is carried by the enzyme.

During phase III, the conjugates formed in Phase II are removed from the cell with the help of ATP. Phase II metabolites have increased hydrophilicity and molecular weight and generally cannot diffuse across the phospholipid membrane barrier [32]. Therefore, Phase III xenobiotic transporters are needed; these excrete hydrophilic conjugates containing anionic groups that act as affinity tags for various membrane carriers. These carriers belong to the following two main groups: ATP binding cassette (ABC) transporters, including the multidrug resistance protein (MRP) family, and solute carrier transporters (SLC) [32]. A key component in this process is the glutathione S-conjugate export pump (GS-X pump), which operates in an ATP-dependent manner [44].

The genes responsible for encoding the drug metabolising enzymes and transporters can vary considerably in populations, which can influence drug absorption and elimination, potentially increasing the risk of therapeutic failure or adverse effects [45,46,47].

There is a need to better understand the interactions, induction, and genetic variability of the metabolic enzymes involved in phases I and II, as this knowledge can be used to predict safe therapeutic dosing and conduct more effective risk assessments of chemicals.

In addition, the course of biotransformation can be affected by various biological and environmental parameters [48,49]. The most important being age, sex, genetic background and mutation, and diet. Studies have found that, in newborns, the enzymes involved in the biotransformation reaction demonstrate 20–50% of the activity of those in adults. In addition, lower CYP1A2 and CYP2E1 activity has been noted in women than men. Finally, the choice of diet can influence the activity of biotransformation enzymes.

## 3. Dangerous Interactions with Food (FDIs) and Herbs (HDIs) During Stage I of Drug Biotransformation

**Figure 4:**
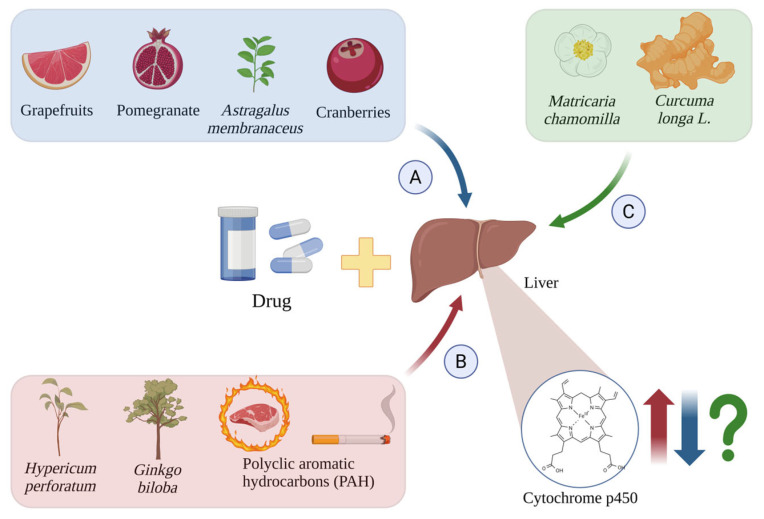
Induction (**A**), inhibition (**B**), and unknown effects (**C**) of CYP450 isoforms by drugs and other chemical substances. The red arrows indicate cytochrome p450 induction by *Hypericum perforatum* [54], *Ginkgo biloba* [55], and PAH [56,57]; the blue arrows indicate their inhibition by grapefruit [58], pomegranate [59], cranberries [60], and *Astragalus membranaceus* [61]; and the green arrows and green question mark show the unknown effects induced by *Matricaria chamomilla* [62] and *Curcuma longa* L. [63,64]. Created in BioRender.com. Agreement number WY28BF4JE7 dated 27 May 2025.

The activity of CYP450 enzymes can be altered by chemical compounds ingested from food and herbs. Such changes influence the process of drug metabolism and thus its plasma levels [50] (Figure 4).

Fruit juices contain a wide range of phytochemicals that can interact with drugs. These interactions are of clinical significance if they increase or decrease systemic drug exposure, resulting in suboptimal pharmacological effects or potential drug toxicity [36]. The mechanisms underlying these interactions are primarily associated with the interaction between phytochemicals and CYP450 enzyme activity. CYP-mediated monooxygenase reactions play a crucial role in FDIs and HDIs. In particular, grapefruit juice is known to interact with approximately 85 different drugs [51], which has been attributed to its inhibitory effect on cytochromes. Of the liver cytochromes, CYP3A is the most abundant, being responsible for the metabolism of approximately 73% of drugs [11].

Grapefruit juice has been found to intensely inhibit intestinal CYP3A4; as such, it can interact with a wide range of medications, and its consumption can result in elevated drug levels and an increased risk of side effects. The interactions between grapefruit juice and drugs are unpredictable and can differ depending on the individual, the medication, and the dosage [52].

One study examined the inhibitory effects of commercially available fruit juices on midazolam 1-hydroxylation, a marker of CYP3A, using pooled human liver microsomes. Juices from black raspberry, black almond, plum, wild grape, white grapefruit, pomegranate, and orange were tested. The degree of inhibition of CYP3A4 by individual juices was found to depend on both the type and the quantity of juice used. The most pronounced inhibition of CYP3A activity was observed for grapefruit juice, followed by black mulberry, wild grape, pomegranate, and black raspberry. Additionally, all fruit juices demonstrated lower IC50 values after preincubation with microsomes in the presence of an NADPH-generating system, suggesting a mechanism-based inhibition similar to that of grapefruit juice. The findings confirm that, like grapefruit juice, various commercially available fruit juices may also have the potential to inhibit CYP3A4 [53].

The juice of *Punica granatum* L. (pomegranate) contains substantial amounts of polyphenols, primarily tannins such as ellagitannin, punicalagin, and punikalin, as well as various flavonoids including anthocyanins, flavan-3-ols and flavonols. Pomegranate juice has been found to influence the bioavailability of *inter alia* warfarin, buspirone, nitrendipine, metronidazole, saquinavir, and sildenafil by reducing intestinal CYP3A4 and CYP2C9 activity [65]. CYP activity is also affected by betanin, the main pigment of beet (*Beta vulgaris* L.), which also exhibits anti-inflammatory, antioxidant, and anticancer properties. Lim et al. (2023) report that betanin inhibits CYP3A4 activity in a dose-dependent manner (IC50 = 20.97 µM) and recommend caution when using betanin in combination with other drugs that are substrates for CYP3A4 [66]. Examples of CYP interactions with fruit juices are presented in Table 1.

Chen et al. present a review of randomised controlled trials examining drug interactions induced by fruit juices, excluding grapefruit juice. Their findings include a number of positive interactions, as follows: orange juice significantly increased iron absorption from iron fumarate; lemon juice improved hepatobiliary excretion of and 99mTc-tetraphosmin; pomegranate juice reduced oxidative stress and inflammation induced by intravenous iron administration; cranberry juice increased the rate of bacterial eradication in women receiving omeprazole, amoxicillin, and clarithromycin for *Helicobacter pylori*; berry juice significantly increased the efficacy of etanercept and reduced its side effects; and lime juice increased the efficacy of the antimalarial drugs artemether and camochin [80]. Some interactions resulted in reduced drug bioavailability and lower efficacy, depending on the volume of juice consumed as follows: apple juice with fexofenadine; orange juice with celiprolol, montelukast, fluoroquinolones, and alendronate; pomelo juice with sildenafil; and grape juice with cyclosporine. Others were characterised by increased bioavailability as follows: Seville orange juice with felodipine, pomelo juice with cyclosporine, and orange juice with aluminium-containing antacids [80]. The authors indicate that, unlike grapefruit juice, which strongly inhibits CYP3A4, most fruit juices generally do not cause serious adverse interactions with CYP, although occasional cases have been reported. They also report that of juice–drug interactions can be influenced, *inter alia*, by the volume of juice consumed, the type and variety of fruit, the time between juice consumption and drug administration, and the polymorphisms in the genes encoding specific enzymes or transporters [80].

## 4. Inhibition of CYP450 by Compounds Found in Grapefruit and Its Juices

The grapefruit juice components responsible for FDIs include furanocoumarins such as bergamottin, 6′,7′-dihydroxybergamottin, and paradisins, as well as flavonoids [51]. Bergamottin inhibits several CYP450 isoforms, including CYP1A2, CYP1B1, CYP2A6, CYP2B6, CYP2C9, CYP2C19, CYP2D6, CYP2E1, CYP3A4, and CYP3A5. Paradisin and 6′,7′-dihydroxybergamottin inhibit the in vitro activity of CYP1A2, CYP1B1, CYP2C9, CYP2C19, CYP2D6, and CYP3A4. Interestingly, different furanocoumarins inhibit CYP3A4 to different degrees in vitro, in the following order: paradisins > 6′,7′-dihydroxybergamottin > bergamottin > bergaptol [67].

**Figure 5:**
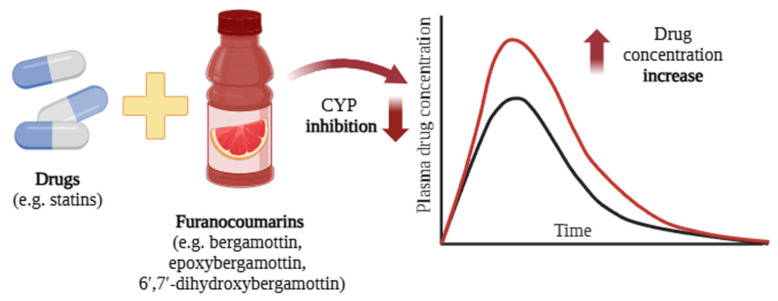
Interactions between grapefruit components and drugs (e.g., statins [57]) lead to an increase in plasma drug concentration [58]. The black curve represents the blood concentration of the drug when administered alone, whereas the red curve depicts the plasma concentration following co-administration with grapefruit or grapefruit juice. Created in BioRender.com. Agreement number UV28BF5ISE dated 27 May 2025.

Thus, exposure the grapefruit juice can increase the concentration of certain drugs (e.g., antihistamines, cyclosporine, and statins) in the blood, thus increasing the risk of side effects. This risk is particularly pronounced for older and more vulnerable individuals [36,81] (Figure 5). A meta-analysis of 51 studies demonstrated that grapefruit juice significantly reduced the area under the curve (AUC) and maximum plasma concentration (C_max_) of aliskiren and celiprolol by approximately 80–90%. In contrast, the AUC and C_max_ of calcium channel blockers were reduced by various degrees when co-administered with grapefruit juice [82].

Grapefruit components impair the function of CYP450 enzymes by being transformed into reactive intermediates that bind covalently to the active site of the enzyme [83]. It has been found that CYP activity can be inhibited by consuming as little as 250 mL of grapefruit juice. This effect lasts for approximately four hours, increasing the bioavailability of the ingested drug for up to 24 h. The time interval between grapefruit consumption and drug intake is critical in preventing this interaction. When grapefruit is consumed less than four hours before taking the drug, the risk of FDI is significantly increased; approximately 10 h after grapefruit consumption, the risk of interaction decreases by 50%, and after one day, the risk drops to 25% [20,36].

The compounds found in grapefruit have been found to react with a total of 85 drugs, including antihistamines, cyclosporine, calcium channel blockers, cisapride, and certain antiviral drugs used in HIV treatment (Table 2). For 43 of these 85 drugs, grapefruit consumption can be life threatening. The elderly population may be at increased risk due to their higher frequency of drug use, as well as their increased consumption of grapefruit juice [51].

Among the compounds found in grapefruit, furanocoumarins play an important role in drug interactions by inhibiting CYP3A4. Guttman et al. found no inhibition of CYP3A4 in two low-furanocoumarin grapefruit varieties, unlike the standard variety. Although bergamottin and 6′,7′-dihydroxybergamottin are weak inhibitors, they are present at high concentrations in grapefruit and hence may have a noticeable effect. The findings indicate that furanocoumarins inhibit CYP3A4 additively when combined with other juice components [92].

### 4.1. Interactions of Compounds Present in Grapefruit and Its Juices with Statins

The interactions between statins and grapefruit juice occur primarily through CYP3A4 inhibition, combined with, to a lesser extent, the inhibition of Pgp and organic anion transport polypeptides.

Changes in plasma drug concentrations resulting from these interactions can enhance the side effects of statins. Therefore, patients taking statins that are substrates for CYP3A4, such as lovastatin, simvastatin, and atorvastatin, are advised not to consume grapefruit juice [58].

A randomised trial with 10 healthy volunteers evaluated the impact of grapefruit juice on statins. Over three days, the participants consumed 200 mL of grapefruit juice or water, followed by a 40 mg dose of simvastatin. The results showed that grapefruit juice increased the AUC of simvastatin by 3.6 times and significantly raised plasma concentrations. Daily consumption of grapefruit juice was linked to a higher risk of side effects, including muscle pain and rhabdomyolysis, i.e., the damage and destruction of human skeletal muscle cells, myocytes [68].

A study evaluated the knowledge of the interaction between statins and grapefruit among statin users attending the outpatient clinic at King Fahd Hospital and community pharmacies, Eastern Province of Saudi Arabia. It was found that 62% of statin users were unaware of any interaction between statins and grapefruit, and only 11% correctly identified the potential interaction effect. Furthermore, a small proportion of patients reported receiving information on drug–food interactions from healthcare professionals, with 11% receiving advice from pharmacists, 21% from doctors, and 6% from nurses [16].

Several cases of rhabdomyolysis have been associated with statin intake, with a key factor being the consumption of significant amounts of grapefruit or grapefruit juice [93,94].

A 40-year-old woman was admitted to the emergency department for bilateral weakness of the lower extremities. Rhabdomyolysis was diagnosed, as evidenced by a significant increase in serum myoglobin levels (6453 g/L), creatine kinase activity (12,640 U/L), aspartate aminotransferase activity (623 U/L), and alanine aminotransferase activity (700 U/L). The patient had a history of hypercholesterolemia and therefore received simvastatin at a dose of 80 mg per day at bedtime. Statin therapy was suspected to be the cause of rhabdomyolysis, leading to the discontinuation of simvastatin. The patient was discharged in excellent physical condition six days after admission. A review of the medical history revealed that 14 days before hospitalisation, i.e., four days before the onset of symptoms, the patient had begun consuming one fresh grapefruit daily for breakfast, which may have contributed to the development of rhabdomyolysis [93].

However, some studies suggest the risk associated with grapefruit juice is low compared to the significant benefits of preventing heart disease, and they argue that grapefruit juice should not be contraindicated for those taking statins. Lee et al. [95] report that a daily glass of grapefruit juice increased blood levels of simvastatin and lovastatin by approximately 260% when consumed with the drug and by approximately 90% if taken 12 h apart. Due to their moderate lipid-lowering effects, lovastatin and simvastatin are not commonly used today and have been replaced by atorvastatin. However, atorvastatin has a long half-life, which makes the timing between statin administration and drinking grapefruit juice consumption less critical. In the study, atorvastatin blood levels were found to increase by approximately 80% when taken with grapefruit juice (at the same time or 12 h after juice intake) [95].

### 4.2. Interactions Between Compounds Found in Grapefruit and Grapefruit Juice with Antihypertensive Drugs

#### 4.2.1. Interaction with Felodipine

A 1989 study on the effect of ethanol on the calcium channel blocker felodipine found the drug to interact with grapefruit juice, which was used to mask the taste of ethanol. The study included 10 patients with untreated borderline hypertension. Those who consumed felodipine with grapefruit juice had a stronger drug response, including increased side effects such as hypotension and tachycardia. In addition, their plasma felodipine levels were over five times higher than those who took the drug with water [96].

In the study by Lown et al., consumption of a single glass of grapefruit juice increased the mean AUC and Cmax of felodipine to 267% and 345%, respectively, compared to administration with water. When grapefruit juice was taken three times daily with meals over a 5-day period, felodipine AUC and Cmax rose further to 345% and 538%, indicating the cumulative effect of the juice [97]. The combination of grapefruit juice and felodipine was associated with a reduction in blood pressure and an increased incidence of orthostatic hypotension.

#### 4.2.2. Interaction with Nifedipine

Adigun et al. describe the case of a 59-year-old man who had been treated for hypertension for 13 years and took nifedipine daily [98]. The man presented to the doctor with swelling of the face and ankles that had persisted for six months. During this time, he had been consuming about 400 mL of grapefruit juice per day. An examination of the patient indicated a possible interaction between grapefruit juice and nifedipine, and the patient was recommended to stop consuming grapefruit juice. A follow-up check-up was scheduled two weeks later. It is believed that the grapefruit juice prevented the metabolism of nifedipine by inhibiting CYP3A4 activity [99].

#### 4.2.3. Interaction with Verapamil

A 42-year-old woman was admitted to the hospital with complete heart block, a ventricular escape rhythm of 34 beats per minute, low blood pressure, and respiratory failure. She had accidentally taken two extra verapamil SR 120 mg tablets within six hours of the first dose. After treatment with respiratory support, pacing, vasopressors, and calcium chloride, her verapamil levels were five times above the therapeutic limit. Additionally, the metabolite norverapamil was elevated. The patient improved following treatment. It was found that, due to nausea, she had ingested significant amounts of grapefruit juice in the days before her admission, consuming an estimated three to four litres over the week leading up to her hospitalisation [100].

The authors attribute the observed increase in plasma concentration to the altered pharmacokinetics of verapamil caused by long-term grapefruit juice consumption; this increased the bioavailability of verapamil by inhibiting CYP3A4 and Pgp activity. Verapamil undergoes extensive first-pass metabolism, with only 20–35% of the drug reaching the systemic circulation. Tracy et al. report that CYP3A4, CYP3A5, and CYP2C8 play an important role in verapamil metabolism [101], and Pgp is inhibited by both grapefruit juice and verapamil.

Controlled studies have shown that the amount of grapefruit juice consumed plays a significant role in its interaction with verapamil. Zaidenstein et al. studied ten patients with hypertension who were chronically treated with verapamil [101]. It was found that a single dose of the drug with 200 mL of grapefruit juice one hour before breakfast had a significant effect on the pharmacokinetics of the drug [102].

#### 4.2.4. Interaction with Amiodarone

A case report describes an 83-year-old woman with a history of myocardial infarction and paroxysmal atrial fibrillation who came to the emergency department with postprandial syncope and palpitations. The patient had been receiving chronic amiodarone therapy and reported consuming 1 to 1.5 L of grapefruit juice daily. It was deduced that the high level of grapefruit juice consumption inhibited amiodarone metabolism, and that this was responsible for the increased pro-arrhythmic effects of the drug [103]. After a four-day hospital stay, the patient was discharged with instructions to avoid excessive consumption of grapefruit juice. As amiodarone is metabolised by CYP3A4 [104], this may have been the route by which the grapefruit juice inhibited amiodarone metabolism, leading to bradycardia and a marked prolongation of the QT interval, which was associated with ventricular arrhythmia.

### 4.3. A Case of Purpura Associated with the Inhibition of Cilostazol Metabolism by Compounds Present in Grapefruit Juice

In 2007, Taniguchi et al. describe a case involving a 79-year-old male patient who developed purpura due to the simultaneous consumption of cilostazol, aspirin, and grapefruit juice [105]. The purpura resolved after discontinuing grapefruit juice while maintaining the other medications. Cilostazol, a reversible platelet aggregation inhibitor, is metabolised mainly by CYP3A4, and its inhibition by grapefruit juice results in increased drug concentrations in the plasma [106]. In this case, purpura was likely caused by the elevated levels of cilostazol in the bloodstream.

### 4.4. Effect of Grape Juice on Docetaxel Drug Metabolism in Oncology Patients

Valenzuela et al. reported a case involving a 52-year-old woman with oesophageal squamous cell carcinoma who was receiving treatment with docetaxel and was consuming grapefruit juice on a daily basis. Docetaxel is eliminated primarily through CYP3A4-dependent metabolism [107]. Consuming 250 mL of grapefruit juice daily reduced plasma clearance of docetaxel from 13.2 L per hour to 36.7 L per hour. This increased drug exposure and resulted in hematologic toxicity, most notably, an approximate 71% reduction in neutrophil count [108].

### 4.5. Effect of Grape Juice on Methadone Drug Metabolism in Patients with Chronic Pain

A 51-year-old man was discovered unresponsive, showing signs of hypoxia, bradypnea, and constricted pupils. His respiratory condition improved after multiple boluses of naloxone with an infusion. Upon regaining consciousness, the patient revealed that he had been receiving 90 mg of oral methadone per day as part of an opioid treatment programme and denied using any other substances. He also reported consuming approximately 500 mL of grapefruit juice daily for three consecutive days prior to his presentation. He was subsequently discharged with advice to discontinue grapefruit juice consumption [109].

Methadone is a synthetic mu-opioid receptor agonist commonly prescribed for chronic pain and opioid dependence. It is metabolised by various CYP isoenzymes, mainly CYP3A4, CYP2B6, and CYP2D6. Consumption of inhibitors such as grapefruit juice can lead to elevated blood levels of methadone, which can result in significant opioid toxicity [110].

### 4.6. Effect of Masked Grapefruit in Orange Marmalade on Tacrolimus Metabolism in Post-Transplantation Patients

A physician experienced nephrotoxicity resulting from a clinical interaction between tacrolimus and marmalade with grapefruit extract [111]. This case demonstrates that prolonged cooking, as in marmalade, does not eliminate the risk of drug interactions with grapefruit. It also indicates that even healthcare professionals, who are fully aware of the risks of drug–food interactions, may still unknowingly experience consequences related to them.

Approximately four months after a transplant, a patient, who was a physician, began to experience anxiety, fever with persistent trembling, difficulty writing, and visual problems. Eventually, he started to feel severe pain in his left chest and was hospitalised. Renal dysfunction was indicated by a very high serum creatinine level, of up to 174 μmol/L. The total blood tacrolimus concentration was found to have increased to 55.4 ng/mL. As a result, tacrolimus therapy was discontinued, and the patient was transferred to the Liver Transplant Unit. Tacrolimus blood levels were monitored daily; after three days, when they had returned to the therapeutic range, therapy was resumed at a dose of 1 mg twice daily.

The medical history indicates that just before the incident, the physician had consumed more than 1.5 kg of an orange marmalade over the course of a week; it had been prepared by a grateful patient, which had substituted half of the marmalade with grapefruit, as bitter oranges were not available at that time. It is hence highly likely that certain components of the grapefruit had interacted with the drug; most probably the components had inhibited CYP3A4 and CYP3A5, which metabolise tacrolimus [112].

## 5. Inhibition of CYP450 by Compounds in Cranberry Juice, Goji Fruit Juice, and Pomegranate Juice

### 5.1. Description of Cases of Warfarin Interaction with Components of Cranberry Juice

Warfarin, a coumarin derivative and vitamin K antagonist, is commonly used in medicine as an oral anticoagulant. Its pharmacological effects can be mainly attributed to its S-enantiomer, which is metabolised by CYP2C9. In contrast, the R-enantiomer is metabolised by CYP1A2 and CYP3A4 [113]. Consequently, drugs and other substances that inhibit or induce CYP2C9, CYP1A2, and/or CYP3A4 can alter warfarin exposure, potentially affecting the international normalised ratio (INR).

Cranberry extracts are frequently used to prevent urinary tract infections. However, the juice contains various antioxidants, such as flavonoids, which are known to inhibit certain CYPs [114]. For example, the metabolites appear to particularly inhibit CYP2C9, the primary enzyme responsible for the metabolism of S-warfarin, potentially increasing the risk of bleeding. Cranberry juice should not be consumed with warfarin and should be strictly avoided [115].

A 71-year-old man was admitted to the Northern Arizona VA Health Care System medical centre due to haemoptysis, hemiplegia, and shortness of breath, which had persisted for two days. The patient had been taking 18 mg of warfarin per week for atrial fibrillation as prophylaxis against ischemic stroke. No changes in medication, diet, or general health were observed during this period. Two weeks before admission, the patient had begun drinking 680 grammes of cranberry juice daily as a source of vitamin C. The postadmission laboratory results revealed a decrease in haemoglobin (8.8 g/dL, compared to 15.3 g/dL at baseline), as well as prolonged INR (>18) and prothrombin time (>120 s). The anaemia and prolonged clotting times were attributed to the interaction between warfarin and cranberry juice. This case strongly suggests that cranberry juice potentiates the activity of warfarin [60].

Another case involved a 70-year-old male who was treated with cephalexin for chest infection. Soon after, he experienced a significant decrease in appetite for two weeks and consumed almost no food except cranberry juice, in addition to his regular medications (digoxin, phenytoin, and warfarin). Six weeks after initiating cranberry juice consumption, the patient was hospitalised with an INR greater than 50. Prior to this event, his INR had been within the therapeutic range. The patient later succumbed to gastrointestinal and pericardial haemorrhage [116].

Another patient taking warfarin for a mitral valve prosthesis experienced an elevated INR of 11 two weeks after regularly consuming cranberry juice. The patient also exhibited complications associated with postoperative bleeding, which was attributed to a potential interaction between the medication and the juice. The conclusion was that warfarin-treated patients should restrict their consumption of cranberry juice [116].

In another case, a 46-year-old woman taking 56 mg of warfarin weekly had an average INR of 2.0 over the four months prior to the incident. However, her INR increased to 4.6 after consuming approximately 1420 mL of cranberry juice cocktail per day for two days; this value then decreased to 2.3 after a 14-day period without cranberry juice. For the following three months, while continuing to take warfarin at the same dosage, her average INR was 2.1. At her next visit, after consuming approximately 2 litres of cranberry juice cocktail per day for 3–4 days, her INR increased to 6.5. These data strongly indicates an interaction between warfarin and cranberry juice [116].

### 5.2. Interaction Between Warfarin and Components of Lycium barbarum L. (Goji) Fruit

*Lycium barbarum* L. is a Chinese plant believed to have tonic effects on various organs and is commonly used in medicine. It produces small red fruits called goji berries, which are highly valued for their nutritional properties and have been known in China for over 2000 years. Today, goji berries are widely incorporated as a key ingredient in health foods throughout the world. They are believed to play an important role in the prevention and treatment of various chronic diseases, including diabetes, hyperlipidaemia, thrombosis, immune deficiency, cancer, hepatitis, and male infertility [117]. Goji berries are a source of phenolic compounds, including phenolic acids, flavonoids, phenylpropanoids, coumarinsand lignans, and their derivatives [118]. *Lycium barbarum* (goji) juice has been shown to strongly inhibit most major CYP450 enzymes. The polyphenolic fraction of the fruit exhibits a more substantial effect than the polysaccharide fraction, with IC50 values of 0.119 mg/mL for CYP3A4 and 0.048 mg/mL for CYP2C9. For the polysaccharide fraction, the respective values were 2.244 mg/mL (CYP3A4) and 4.094 mg/mL (CYP2C9) [119]. Elsewhere, goji juice caused a 75% inhibition of the main CYP compared to cold/hot goji extract, which induced less than 30% inhibition. In addition, 80% ethanol extracts exhibited more potent inhibition of CYP2C9 and CYP2C19, i.e., more than 90% [120]. Considering that CYP2C9 is the primary enzyme responsible for the metabolism of S-warfarin, this is an important finding [113].

Warfarin has been found to interact with Gouqizi wine. One such case concerned a 65-year-old Chinese man who had been treated with warfarin for approximately two years following mechanical heart valve implantation, with no comorbidities [121]. The patient followed his doctor’s instructions, and his INR was maintained within the therapeutic range. On 10 November 2013, the patient developed haematuria and was admitted to hospital. Urinalysis confirmed the presence of haematuria, with an abnormal red blood cell count of 33,201.5/L (normal range: 0–23/L) and an INR of 3.84. The patient reported feeling well and denied taking any additional medications or making changes in his diet. He acknowledged consuming 20 mL of Gouqizi wine on the evening of November 9. The patient was aware that wine could influence the effects of warfarin by increasing its concentration in the blood, thus increasing the risk of bleeding. However, since he had not observed any bleeding symptoms after consuming 60 mL of a different type of wine (not containing Gouqizi), he decided to try this alternative wine. The patient was advised to discontinue the use of Gouqizi wine, and his INR returned to normal. The report notes that a large dose of Gouqizi (more than 6–12 g) can significantly increase the risk of bleeding during Warfarin therapy.

Another case describes a female patient whose warfarin levels and INR were influenced by consuming concentrated goji berry herbal tea [122]. A 61-year-old Chinese woman experienced an increase in her INR from a range of 2–3 to 4.1 after drinking three to four glasses of concentrated herbal tea daily for four days. After discontinuing herbal tea, the INR value returned to 2.4 after seven days, and her seven consecutive INR values remained within the range of 2.0–2.5. It is possible that the goji berry tea inhibited cytochrome activity, particularly that of CYP2C9 [119], which is primarily responsible for the metabolization of warfarin [120].

### 5.3. Interaction Between Sildenafil and Pomegranate Juice (Punica granatum)

Pomegranate components inhibit the activities of CYPs such as CYP3A4 and CYP2C9 and can affect the intestinal and liver metabolism of drugs mediated by CYP3A4 and CYP2C9 [65]. Sildenafil is mainly metabolised by CYP3A4 and, to a lesser extent, CYP2C9 [123].

Sildenafil citrate, a potent and selective inhibitor of cyclic guanosine monophosphate (cGMP), is commonly used to treat erectile dysfunction [124]. The literature describes cases where the concurrent use of sildenafil and pomegranate juice (*Punica granatum*) was associated with priapism, an emergency condition that requires immediate intervention to alleviate complications and minimise the risk of impotence. One case involved a 46-year-old man who came to the emergency room with a persistent and painful penile erection that had lasted five hours after sexual intercourse with his wife [60]. Upon interview, the patient revealed that he had been prescribed sildenafil for psychogenic erectile dysfunction; however, an alternative medicine practitioner recommended that he drink 200 mL of pomegranate juice daily to enhance vigour and vitality. Upon consuming pomegranate juice together with a 50 mg dose of sildenafil for the first time, the patient achieved an erection within 15 min, which continued even after ejaculation. He was not taking any other medications or herbal supplements at the time.

The priapism was resistant to painkillers, ice packs, and subcutaneous terbutaline. Consequently, the patient was treated with epinephrine and 2% lidocaine, leading to complete decompression within 15 min. The patient was discharged without complications and advised to refrain from drinking pomegranate juice while using sildenafil. He continued to take 50 mg doses, which caused erections that resolved immediately after orgasm [59].

## 6. Inhibition of CYP by Compounds in Selected Herbs

Patients often assume that herbal medicines are free of adverse effects and are increasingly turning to these remedies as alternatives or complements to conventional therapy. However, medicinal plants can pose significant risks, either independently or in combination with other pharmacological agents. The adverse effects of herbal products vary in severity and can include mild to severe reactions such as allergic reactions, skin rashes, headaches, nausea, vomiting, and diarrhoea [125]. Over the past few decades, the use of herbal preparations among patients has increased significantly. Approximately 20–35% of individuals receiving conventional pharmacotherapy consume herbal products at the same time [126]. Consequently, concerns about possible interactions between the active constituents of herbal remedies and conventional drugs are well justified [127]. This issue is particularly relevant in the elderly population, who are more likely to multiple medications and consume herbal supplements. A survey of 400 older adults aged over 65 years of age found that of the 155 completed the questionnaires (response rate = 38.8%), 33.6% reported the concurrent use of herbal preparations and dietary supplements along with prescription medications. Women were more likely than men to combine the treatments. Among the respondents, 16 individuals (32.6%) were identified as at risk of potential adverse drug interactions [128].

Few cases of the adverse effects associated with medicinal herbs have been reported. Nevertheless, such interactions represent a significant safety concern, particularly for medications with narrow therapeutic indices, such as warfarin [122,129,130], which can cause severe side effects that can be life threatening [131,132].

A 2018 review of adverse reactions resulting from HDI in patients identified 49 case reports and two observational studies, covering a total of 15 cases of adverse effects. The most common disease entities in the study population were cardiovascular diseases (30.6%), oncological diseases (22.45%), and kidney transplant cases (16.32%). The predominant drugs used were warfarin, alkylating agents, and cyclosporine. The HDIs resulted in clinically significant adverse reactions of varying severity. Such severe interactions can reduce treatment efficacy or increase the risk of toxicity, which consequently leads to increased healthcare costs and can involve hospitalisation or prolonged hospitalisation [126].

A 2022 review examined the risks posed by various medicinal plants used in the treatment of inflammatory diseases such as rheumatoid arthritis (RA), inflammatory bowel disease (IBD), and osteoarthritis. The analysis indicated that St. John’s wort, cannabis, green tea, and echinacea possess a high potential for HDI and may interfere with several conventional drugs. Flaxseed, ginger, meadowsweet, psyllium, valerian, and willow bark were identified as having moderate interaction potential. In contrast, boswellia, chamomile, cranberry, devil’s claw, garlic, ginseng, milk thistle, peppermint, and turmeric generally exhibited a low interaction potential; however, possible interactions with cyclosporine and tacrolimus can occur via CYP enzymes or P-glycoprotein (P-gp) mechanisms. Finally, artichoke, ash leaf, blackcurrant leaf, English plantain, fennel, lemon balm, linden flowers, nettle herb, rose hip, saw palmetto, soybean, and wormwood did not demonstrate any reported interactions or only a very low risk [133].

Terpenoids, phenylpropanoids, flavonoids, alkaloids, and quinones derived from herbs have been identified as natural inhibitors of P450 enzymes [134], thus influencing drug metabolism. In particular, antibiotics, oral hypoglycaemic agents, and anticonvulsants exhibit synergistic interactions with herbal compounds [135].

Certain herbal preparations used in Africa have also been found to exert herb–drug interactions. Amaeze et al. evaluated the potential risk of HDIs for the following five medicinal plants: *Vernonia amygdalina*, *Ocimum gratissimum* L., *Moringa oleifera*, *Azadirachta indica*, and *Picralima nitida*, using in vitro tests [136]. These plants are often used to treat diabetes and other conditions, in Nigeria; however, little is known regarding their potential impact on drugs. The methanolic extracts of *O. gratissimum* reversibly inhibited the enzymes CYP 1A2, 2C8, 2C9, and 2C19 (IC50: 6.21 µg/mL, 2.96 µg/mL, 3.33 µg/mL, and 1.37 µg/mL, respectively). Furthermore, the methanolic extract of *V. amygdalina* inhibited the activity of CYP2C8 (IC50: 5.71 µg/mL); methanolic and aqueous extracts inhibited the activity of CYP2D6 (IC50: 1.99 µg/mL and 2.36 µg/mL, respectively), while the methanolic extract of *A. indica* inhibited CYP 3A4/5, 2C8, and 2C9 (IC50: 7.31 µg/mL, 9.97 µg/mL, and 9.20 µg/mL, respectively) [136]. The table below presents examples of drug interactions in humans that are potentially clinically significant due to herb–drug interactions (HDIs) (Table 3).

### 6.1. Interaction of Warfarin with Components of Chamomile (Matricaria chamomilla)

Chamomile (*Matricaria chamomilla*) is a versatile plant with applications in treatment, cosmetics, and nutrition [142]. Chamomile extracts and tea are commonly used as herbal remedies for minor ailments [143]. Like many other herbs, chamomile has been shown to predominantly inhibit the cytochrome CYP1A2 isoenzyme [144]. Numerous studies have shown that various components of natural plants, particularly those from herbal medicines, can inhibit CYP2C9 activity; CYP2C9 metabolises the S enantiomer of warfarin, which is responsible for its anticoagulant activity [145]. Although chamomile is considered a weak CYP2C9 inhibitor [146], evidence suggests a possible warfarin–chamomile interaction, with some researchers highlighting the increased risk of bleeding when chamomile tea is consumed concurrently with anticoagulants [147].

In one case, a 70-year-old woman was hospitalised for multiple internal haemorrhages while receiving warfarin treatment after the use of chamomile products (tea and body lotion) to alleviate upper respiratory symptoms. This case represents the first documented instance of an interaction between warfarin and *M. chamomilla* [62]. The patient presented dyspnoea during exertion, bilateral foot swelling, and petechiae in the perineal area, lower abdomen, and over the left hip. She had previously attempted to alleviate swelling in her feet by applying chamomile skin lotion (one teaspoon per foot, 4–5 times a day). In addition, she consumed 4–5 cups of chamomile tea daily while using camphor lotion to ease chest congestion. Both chamomile products were commonly used once or twice daily. After receiving treatment, she was discharged with stable haemoglobin levels and an INR of 2.5, with scheduled follow-up at a cardiology and anticoagulation clinic. This case highlights the importance of educating patients about the potential risks associated with the simultaneous use of chamomile products and warfarin therapy [62].

A recently published randomised, placebo-controlled crossover study of 12 healthy subjects evaluated whether chamomile consumption affects clotting tests mediated by coumarin-like substances. It was found that the seven-day consumption of chamomile in the form of tea (three tea bags, three times per day), capsules (three times daily), or a placebo capsule (three times daily) did not increase clotting time. These results suggest that it may not be necessary to avoid perioperative chamomile intake in patients taking warfarin. Ingestion of chamomile tea or extract capsules was not found to have any deleterious effect on prothrombin time or any of the prespecified secondary endpoints of anticoagulation [148].

### 6.2. Interaction of Nifedipine with the Herbal Product SHENG Mai-San

Nifedipine, a first-generation calcium channel blocker, is widely used to manage hypertension. It undergoes metabolism by the enzymes CYP3A4 and CYP3A5, forming inactive metabolites [149]. The potential for herbal interactions with nifedipine, particularly through the inhibition of CYP3A, has significant clinical relevance. Sheng Mai-San, a traditional Chinese herbal formulation, is frequently prescribed in Asian populations for the treatment of cardiovascular diseases [150].

A retrospective cohort study by Wang et al. examined the effect of Sheng Mai-San on nifedipine and felodipine treatment in 4894 hypertensive patients [151]. It was found that patients who received Sheng Mai-San together with their medication experienced a higher incidence of headaches (92.70 per 1000 person-years) than those who did not receive Sheng Mai-San (51.10 per 1000 person-years). Similarly, pharmacokinetic studies of nifedipine in rats found three-week treatment of Sheng Mai-San increased systemic exposure to nifedipine by almost twofold and decreased nifedipine clearance by 39%. Of the herbal constituents present in Sheng Mai-San, schizandrin B, schizandrin A, and methylphiopogonanone A were found to inhibit oxidation activity in the liver and intestinal microsomes of rats, as well as human CYP3A4. Furthermore, methylphiopogonanone A appears to be a time-dependent inhibitor of CYP3A4 [152].

Three-week Sheng Mai-San administration resulted in increased plasma levels of nifedipine in rats. Additionally, patients undergoing long-term treatment with nifedipine/felodipine along with Sheng Mai-San experienced a higher frequency of headaches. This result is probably due to the herbal preparation inhibiting CYP3A4, resulting in higher systemic drug levels.

### 6.3. Effect of Herbal Substances on Tacrolimus Levels

Tacrolimus is a potent immunosuppressant commonly used in liver, kidney, heart, and marrow transplantation to prevent transplant rejection in both adults and children, particularly when other immunosuppressive therapies are ineffective. The drug is mainly metabolised by CYP3A4 and CYP3A5 [112], with CYP3A5 being the dominant enzyme involved in tacrolimus metabolism [143]. Consequently, the concurrent use of drugs or foods that inhibit CYP3A4 and CYP3A5, such as turmeric [152,153,154], can significantly influence tacrolimus blood concentrations.

Some significant interactions have been reported between tacrolimus and various herbal extracts. The CYP isoenzymes CYP3A4 and CYP3A5 and Pgp are involved in tacrolimus bioavailability. The bioavailability of tacrolimus has been found to increase when administered concurrently with grapefruit juice, *Schisandra Michx.*, berberine, turmeric, pomegranate juice, pomelo, or ginger in both human and animal models. These effects have been attributed to their potential to inhibit CYP3A4 [154]. In contrast, tacrolimus bioavailability is reduced when co-administered with St. John’s wort (*Hypericum perforatum*), rooibos tea, and boldo in human models, probably due to the induction of the CYP450 system [152].

A review of 65 studies of HDI found herbal substances to inhibit the activity and hence the therapeutic efficacy of cyclosporine, representing approximately 27% of the studied cases, and tacrolimus, approximately 19%. The highest number of interactions was noted between *Hypericum perforatum* and cyclosporine and tacrolimus. In contrast, *Schisandra sphenanthera* (citronella) interacted most frequently with tacrolimus, resulting in increased bioavailability. In particular, most of the reviewed studies were conducted in animal models [155].

Hence, Miedziaszczyk et al. emphasise that patients initiating tacrolimus therapy should be advised against excessive consumption of grapefruit, pomelo, mandarin, pomegranate, ginger, turmeric, and green tea [156]. The authors also suggest that *Panax ginseng*, green tea, and *Schisandra sphenanthera* (citronella) may have protective properties which play a significant role in mitigating the side effects of tacrolimus, and that if consumed in moderate amounts, they may improve the health of recipients. The authors also highlight that while the aqueous solution of St. John’s wort contains negligible concentrations of hyperforin, the hydroalcoholic extract has sufficient levels to stimulate CYP3A4 activity. Consequently, the co-administration of St. John’s wort extract with tacrolimus is not recommended due to the potential for enzyme induction and the subsequent risk of interactions [156].

#### 6.3.1. Case Report of an HDI Between Tacrolimus and Turmeric Resulting in Acute Nephrotoxicity

Nayeri et al. report the first documented case of a possible food–drug interaction between turmeric and tacrolimus, resulting in the acute nephrotoxicity of the calcineurin inhibitor [63]. A 56-year-old man with a history of orthotopic liver transplantation arrived at the emergency department with worsening oedema and a creatinine level of 4.2 mg/dL. Blood tacrolimus levels were found to have increased to 29.9 ng/mL, despite no changes in the dosing regimen [157]. The patient reported a history of consuming large amounts of turmeric with meals. Subsequently, tacrolimus was discontinued, and the patient was discharged on the fourth day with improved renal function.

#### 6.3.2. Case Report of No Interaction Between Tacrolimus and Turmeric, Curry, and Ginger

A case report found turmeric, curry powder, and ginger to have no effect on the concentration of tacrolimus in plasma [64]. A 70-year-old kidney transplant recipient consumed 10 g of turmeric, curry, and ginger per day for four days during immunosuppressive treatment. No significant changes in tacrolimus plasma concentrations were observed. These findings suggest that the spices have minimal effects on tacrolimus levels, although further research is needed. Interestingly, it was proposed that it may be reasonable for the patient to test the effect of spice consumption on drug levels in patients if a joint decision is made with the physician.

#### 6.3.3. Case Report of the Interaction Between Tacrolimus and Radix Astragalus Membranous

In one reported case, an 8-year-old girl receiving tacrolimus was admitted to hospital with refractory nephrotic syndrome caused by a drug interaction with a herbal preparation containing the root of radix *Astragalus membranous* [61]. The patient’s tacrolimus blood concentration was nearly halved, despite no changes in the dosing regimen. *A. membranous* had been administered in granules of Chinese herbal medicine.

The medical team concluded that the most likely cause of the decrease in tacrolimus levels was the presence of *Astragalus membranous* in the herbal preparation. Indeed, *A. membranous* and its principal bioactive compounds, calycosin and formononetin, have been found to significantly induce the expression of CYP3A4, both in vitro [158] and in vivo in rats [159]. Consequently, the authors propose that induction of CYP3A4 by *A. membranous* led to a reduction in tacrolimus blood levels, resulting in a lack of therapeutic effect on day five of treatment [61].

#### 6.3.4. Effect of *Schisandra sphenanthera* on Tacrolimus and Midazolam

The medical use of *Schisandra sphenanthera* in China dates back thousands of years. Two of its components, schisandrin B and gomisin A, are inhibitors of Pgp, while gomisin C is an inhibitor of CYP3A4 [160,161]. Twelve healthy male volunteers were administered three *S. sphenanthera* capsules twice daily for 13 days. Treatment resulted in a 36.8% increase in tacrolimus tmax, suggesting that *S. sphenanthera* may inhibit CYP3A4 and/or P-gp in the intestine, leading to higher absorption and reduced intestinal metabolism [139]. Similarly *S. sphenanthera* treatment resulted in a 133.3% increase in midazolam tmax and enhanced bioavailability in healthy volunteers [140].

## 7. Induction of CYP450 by Components of Herbs, Fruits, and PAHs

**Figure 6:**
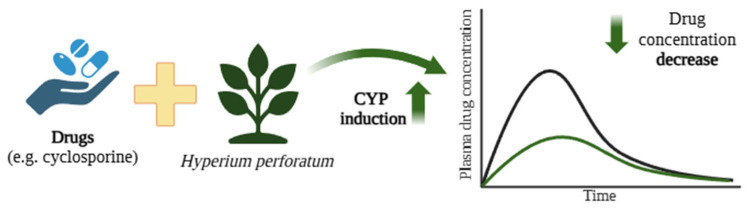
Reduction in cyclosporine concentrations due to CYP P450 induction by components of St. John’s wort (*Hyperium perforatum*) [162]. The black curve represents the blood concentration of the drug when administered alone, whereas the green curve depicts the plasma concentration following co-administration with *H. perforatum*. Created in BioRender.com. Agreement number DE28BF50N6 dated 27 May 2025.

Certain chemical compounds found in herbs, foods (such as grilled meat), or tobacco smoke can induce CYP450, leading to increased drug metabolism (Figure 6). Examples of such interactions, as reported in humans, are summarised in Table 4.

### 7.1. Cases’ Description of Interactions Between Hypericum perforatum and Cyclosporine

*Hypericum perforatum* and its active compounds, including hyperforin and hypericin, have a wide spectrum of medicinal applications, particularly in wound healing, antimicrobial treatments, and mood enhancement [172].

However, *H. perforatum* preparations have been found to demonstrate many interactions with psychotropic drugs, mainly due to their potential to induce CYP isoenzymes, notably CYP1A2, CYP2C9, CYP2C19, CYP2D6, and CYP3A4 [173,174]. These interactions lower the therapeutic plasma concentrations of these drugs. Moore et al. showed that hyperforin, a component with antidepressant effects, strongly binds to the pregnane X receptor (Ki = 27 nM), which regulates CYP3A4 expression. Significant induction of CYP3A4 was observed in primary human hepatocytes treated with St. John’s wort extracts or hyperforin alone [175].

The interaction between the constituents of *H. perforatum* and cyclosporine is illustrated by a case involving a 29-year-old woman who underwent a kidney and pancreas transplant [162]. Cyclosporine, an immunosuppressive drug, is used to prevent transplant rejection and is primarily metabolised in the intestine and liver by CYP enzymes, predominantly CYP3A4, with contributions from CYP3A5 [176]. Cyclosporine has a narrow therapeutic window, and its level is highly susceptible to modulation by substances that influence CYP3A4 and Pgp activity in the liver and small intestine. The patient in this case consumed a *H. perforatum* herbal mixture for four to eight weeks, following which cyclosporine levels became subtherapeutic, which was associated with organ rejection. Four weeks after stopping St. John’s wort, cyclosporine concentrations returned to therapeutic levels. However, the patient developed chronic rejection and ultimately required dialysis [162]. The constituents of *H. perforatum* induced the activity of CYP, including CYP3A4, which led to the faster metabolism of cyclosporine and its subtherapeutic doses, resulting in organ rejection.

A study examined the interaction between *H. perforatum* and cyclosporine in 30 kidney transplant patients [167]. After initiation of *H. perforatum* therapy, a mean reduction of 47% in the cyclosporine level was observed. After stopping St. John’s wort, blood cyclosporine levels increased by an average of 187%. The authors proposed that this effect was likely due to St. John’s wort inducing CYP enzymes in the liver and/or small intestine, enhancing drug metabolism. Another possibility is the induction of the Pgp transporter in the small intestine, leading to the increased export of cyclosporine from the blood into the intestinal lumen [177].

Similar interactions have occurred in the following two patients who had received heart transplants [163]: a 61-year-old who self-medicated with *H. perforatum* for mild depression, and a 63-year-old prescribed the therapy by a psychiatrist for anxiety and depression. The St. John’s wort components were found to induce CYP enzymes and Pgp, leading to reduced plasma cyclosporine levels.

### 7.2. Case Report of the Interaction Between Acenocoumarol and Components of Liquorice (Glycyrrhiza)

A case was described of a 92-year-old female patient diagnosed with atrial fibrillation who was undergoing phenprocoumon (acenocoumarol) therapy to prevent stroke [166]. Her medical history included hypertension, coronary artery disease, type 2 diabetes, mild senile dementia, and renal failure. Despite acenocoumarol treatment, the patient experienced an ischemic stroke. Before the stroke, her INR values were within the therapeutic range of 2–3 but suddenly dropped to 1.25. A retrospective review revealed no significant changes in patient behaviour or adherence to therapy other than the consumption of 1.5 kg of hard liquorice candy on days leading up to the stroke. The abrupt decrease in INR values can be attributed to the effect of liquorice and its compounds on the pharmacokinetics of acenocoumarol. The authors propose that liquorice may have stimulated the activity of CYP3A4 or other CYP enzymes, thus increasing the metabolism of acenocoumarol and reducing its bioavailability in the patient. This mechanism may explain the sudden decrease in the patient’s INR value.

Li et al. report that three species of liquorice commonly used in dietary supplements exhibit varying potential for inhibiting specific CYP isoforms. More specifically, *Glycyrrhiza uralensis* Fisch. ex DC was shown to exert a potent inhibition of CYP2B6, along with the moderate inhibition of CYP2C8, CYP2C9, and CYP2C19 [178]. In contrast, a rat study indicated that liquorice significantly reduced the oral bioavailability of cyclosporine, probably by activating Pgp and CYP3A4 [179]. In contrast, acenocoumarol is metabolised by CYP3A [180], CYP2C9, CYP2C19, and CYP2C8 [181].

### 7.3. Case of Induction of CYP2C9 by Compounds Present in Noni Juices and Reduction in Phenytoin Levels

Kang et al. report a case of a 49-year-old man who had been using phenytoin for epilepsy treatment for over ten years. Despite following medical instructions, the patient exhibited persistent subtherapeutic levels of phenytoin, ranging from low to undetectable, resulting in poor seizure control. It was discovered that the patient consumed noni fruit juice daily; and it may be that components of the juice induced CYP2C9, leading to the accelerated metabolism of phenytoin and a reduced therapeutic effect. Despite these adverse effects, the patient was reluctant to stop consuming the juice due to its perceived health benefits. As a result, clobazam was added to his treatment regimen, and by gradually reducing noni juice intake over six months, the patient’s epilepsy was effectively controlled and no significant seizures were reported for more than a year [77].

It is possible that noni juice may induce CYP enzymes, including CYP2C9, which is responsible for approximately 90% of phenytoin elimination in humans. In contrast the CYP2C19 isoform metabolises only 10% of the drug [182]. Given that noni juice acts as an inducer and phenytoin serves as a substrate of CYP2C9, reduced serum concentrations of phenytoin were observed in the patient after the concurrent administration of noni juice. Furthermore, experiments on rats confirmed that pretreatment with noni juice resulted in a 2.81-fold reduction in phenytoin bioavailability [55].

### 7.4. Case Report of CYP Induction Between Efavirenz and Components of Ginkgo biloba L.

A case report describes a reduction in the therapeutic effect of efavirenz by *Ginkgo biloba* L. due to CYP induction [165]. Efavirenz (EFV), a drug used in combination therapy for human immunodeficiency virus 1 (HIV-1), is metabolised by CYP2B6, and to a lesser extent, by CYP3A [165,183]. Components of *Ginkgo biloba* L. extract have been shown to induce the expression of genes encoding the CYP450 enzyme [55,184].

The patient, a 41-year-old man, had been on a zidovudine, lamivudine, and efavirenz regimen for 10 years following the diagnosis of HIV infection. In June 2010, he experienced viremia at 1350 copies/mL, with a CD4 cell count of 1266 cells/mm^3^. Despite adhering to his medication regimen, the patient revealed that he had recently begun taking 300 mg of *Ginkgo biloba* daily as a dietary supplement. Aware of the potential of the *G. biloba* extract to reduce serum efavirenz concentrations [165], the patient was advised to discontinue the extract. One month after discontinuing herbal supplements, a follow-up blood test revealed a significant reduction in viremia to less than 50 copies/mL. All subsequent HIV tests were negative and the patient did not resume the use of any such supplements [165].

### 7.5. Induction of Monooxygenases by PAHs Present in Tobacco Smoke and Grilled Foods

PAHs are a group of organic compounds characterised by at least two aromatic rings [185]. These compounds are ubiquitously present in the environment, with benzo(a)pyrene (BaP) being the most carcinogenic example [186]. For many individuals, the main sources of exposure to PAH are thermal food processing and smoking [187]. When absorbed into the body, PAHs undergo metabolic transformations. PAHs from tobacco smoke are linked to the induction of CYP450 enzymes, such as CYP1A1, CYP1A2, and possibly CYP2E1 and CYP2B. Consequently, drugs metabolised by these isoenzymes may experience accelerated metabolism after exposure to tobacco constituents [56,188,189,190], and as such, smokers may require higher doses. It is crucial that prescribers and pharmacists are aware of the potential impact of smoking on drug metabolism to prevent complications related to toxicity during smoking cessation.

Examples of drugs that interact with PAHs include the following: bronchodilators: methylxanthines (e.g., theophylline); neuroleptics (e.g., clozapine and chlorpromazine); analgesics (e.g., pentazocine, morphine, and codeine); benzodiazepine derivatives (e.g., alprazolam and diazepam); antidepressants (e.g., amitriptyline, imipramine, and fluvoxamine); anticoagulants (e.g., warfarin); antiarrhythmic drugs (e.g., propranolol and amiodarone); and antidiabetic drugs (e.g., insulin) [191,192].

It is recommended that smokers consult with a doctor when quitting smoking to ensure the appropriate, safe adjustment of medication dose, to prevent any adverse symptoms [193].

For individuals taking medications that interact with PAHs, it is important to be aware of foods that contain them in significant levels. Several strategies can help reduce the PAH content in food. For example, in addition to enhancing flavour and aroma, marinating meat with various spices, such as garlic, containing diallyl disulphide [194], can lower PAH concentrations. Furthermore, frying with fresh oil and steaming or braising can further minimise PAH exposure [185,187].

#### Lack of Effect of Olanzapine Due to the Induction of CYP450 by Compounds Present in Tobacco Smoke

In one case, a 30-year-old man with schizophrenia, who had been smoking cigarettes for more than 10 years, was hospitalised for drug cessation, aggression, and hypotension. During his hospital stay, olanzapine, an atypical antipsychotic from the thienobenzodiazepine derivative group, was administered, which improved his condition [17]. Olanzapine is mainly metabolised by CYP1A2, with a lesser involvement of CYP2D6 [195].

However, the patient was dissatisfied with the hospital smoking ban and resumed smoking 80 cigarettes a day upon returning home. During the following weeks, he returned to the hospital with the same symptoms, despite the continued use of the medication. While in hospital, he reduced his cigarette consumption, and his condition improved. This case highlights the effect of cigarette smoking on the plasma concentration of olanzapine. Smoking cigarettes induces CYP1A2 activity, which in this case impaired the therapeutic effect of olanzapine, despite the patient adhering to the prescribed dose; as such, reducing smoking led to increased drug effectiveness [17].

## 8. Increased Tyramine Levels Due to the Blockage of Monoamine Oxidases by MAO Inhibitors

Tyramine is a hydroxyl derivative of phenylethylamine, which is found in various foods including cheese (e.g., camembert, cheddar, parmesan, and brie), bananas, chocolate, smoked fish, beef liver, and bologna sausage [196,197].

Under normal conditions, tyramine does not accumulate because it is metabolised by monoamine oxidase (MAO). Monoamine oxidases are flavin-containing enzymes that degrade monoamines, such as norepinephrine, serotonin, and tyramine, which act as neurotransmitters. Their activity plays a crucial role in the regulation of the central nervous system [12,198].

MAO exists as two isoenzymes, MAO-A and MAO-B. MAO-A is found predominantly in the gut, liver, and adrenal medullary cells, whereas MAO-B is found mainly in the liver and brain. MAO-A primarily regulates the metabolism of serotonin and norepinephrine, while MAO-B is more involved in the breakdown of dopamine and phenylethylamine. The disruption of tyramine metabolism can lead to *inter alia* increased blood tyramine concentrations, increased intestinal absorption of tyramine, decreased liver metabolism, increased availability of dissolved norepinephrine for release, and the elevated release of norepinephrine and epinephrine from adrenal medullary cells [199].

Tyramine is a substrate for both isoforms of MAO, playing a significant role in the proper functioning of MAO inhibitors [199,200,201]. While these drugs were initially developed to treat depression, their applications have since expanded to include the treatment of affective and neurological disorders, as well as strokes and age-related neurocognitive changes. MAO inhibitors achieve their therapeutic effects by enhancing the activity of monoamine neurotransmitters [202,203]. These inhibitors were the first antidepressants, but their use became limited after the discovery of their negative interactions with certain foods.

Monoamine oxidase inhibitors (MAOI) are classified into three generations as follows: I. Classical MAO inhibitors—nonselective drugs used to treat depression that form irreversible complexes with MAO. An example is phenelzine. II. Selective MAO inhibitors—used in the therapy of Parkinson’s disease, these drugs also form irreversible complexes with MAO. Examples include selegiline (selective for MAO-B) and clorgyline (selective for MAO-A). III. Selective MAO inhibitors with antidepressant properties—these drugs form reversible complexes with MAO. Examples include moclobemide (selective for MAO-A) and lazabemide (selective for MAO-B) [204].

**Figure 7:**
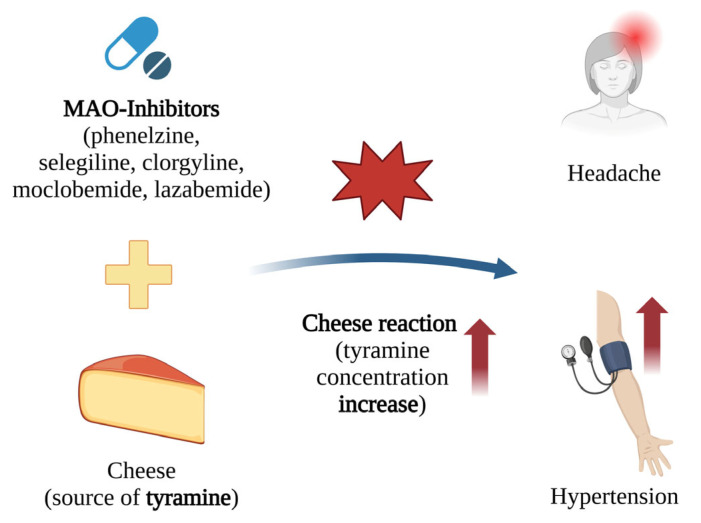
Interaction between foods and MAO inhibitors, where inhibition of tyramine metabolism by MAO inhibitors leads to the health consequences of elevated tyramine levels [197]. The red arrow indicates an increase in blood tyramine levels and blood pressure. An increase in tyramine can cause, among other things, headaches and can potentially trigger a sudden increase in blood pressure, which can lead to a life-threatening stroke [205]. Created in BioRender.com. Agreement number AB28BF3LRB dated 27 May 2025.

When taking MAO-A inhibitors, the consumption of pickled, canned, or fermented foods containing tyramine can cause its accumulation, leading to a dangerous increase in blood pressure. The inhibition of MAO-A significantly reduces the body’s ability to metabolise dietary tyramine, leading to the overstimulation of postsynaptic adrenergic receptors. The ingestion of merely 8–10 mg of tyramine has been found to trigger life-threatening increases in blood pressure [13] (Figure 7).

This phenomenon is commonly known as the “cheese effect” [206], which occurs when people consume foods high in tyramine, such as cheese, while taking MAOI drugs. The cheese effect can cause various symptoms, the most common being headache, vomiting, restlessness, excessive sweating, and pallor. Elevated blood tyramine levels can ultimately cause a sudden increase in blood pressure, which can result in life-threatening stroke [205]. To prevent this adverse effect, a low-tyramine diet is recommended [197,207].

The use of MAO inhibitors in psychiatric treatment has been limited due to the risk of dangerous interactions. Expanding their use into other areas of medicine will depend on further research to eliminate the cheese effect [208,209].

### 8.1. Inhibition of Tyramine Metabolism Due to Phenelzine Intake, the So-Called “Cheese Effect”

A 34-year-old woman being treated for depression with phenelzine, a nonselective MAO inhibitor, consumed cheese three hours post-medication and experienced chest pain, shortness of breath, and a severe headache within an hour [18]. She was taken to the hospital where an EKG revealed a heart attack. After three days of hospitalisation and several tests, she was discharged. The authors suggest that this is a case of myocardial infarction after a MAO inhibitor-induced hypertensive crisis. Indeed, the presence of elevated tyramine levels in patients using MAOI can lead to hypertensive crisis, posing a significant threat to life and health [18].

A 23-year-old man undergoing phenelzine treatment (30 mg in the morning, 15 mg in the afternoon, and 15 mg in the evening) for chronic fatigue syndrome and atypical depression, consumed pizza and parmesan cheese after four months of therapy. Within one hour, the patient experienced sudden pressing chest pain, shortness of breath, severe headaches, palpitations, nausea, and high blood pressure (162/106 mmHg). An electrocardiogram revealed an accelerated nodal rhythm with ST-segment depression, and troponin levels increased to 2.03 ng/mL five hours after admission (normal < 0.4 ng/mL), indicating a myocardial infarction without ST-segment elevation. Coronary computed tomography angiography did not reveal any atherosclerotic lesions or vascular anomalies. Phenelzine was discontinued and the patient was discharged in good condition. After two weeks, an exercise programme was introduced, leading to improvements in fatigue symptoms, and the patient reported no recurrence of depressive symptoms. A follow-up exercise test after three months revealed no signs of ischemia [210].

These cases highlight the risk that a small change in diet or medication can trigger a dangerous interaction, potentially resulting in far-reaching effects.

### 8.2. Intracranial Haemorrhage Following the Use of Tranylcypromine and Beer

The literature also reports a case of acute cerebral haemorrhage in a 47-year-old woman taking an MAO inhibitor, tranylcypromine, after drinking 500 mL of regular beer. Both low-alcohol and non-alcoholic beers contain similar amounts of tyramine [211]. A study of 13 types of non-alcoholic beers found the highest tyramine level in Baltika beer (111.34 ± 8.19 μg/mL) and the lowest in Bitmalt beer (8.01 ± 2.09 μg/mL) [212]. It is recommended that patients taking tranylcypromine avoid all types of beer.

## 9. The Role of the Quantity of Drugs, Food Products, and Herbs Consumed in Adverse Drug Interactions

Numerous studies carried out in volunteers have shown that the development of interactions that disrupt the proper level of the drug depends on the dose of the drug taken, as well as the amount of food and herbs consumed.

Food products and herbs are more likely to have an effect on a drug when they are consumed systematically in significant doses. The appearance of rhabdomyolysis was reported in patients taking atorvastatin with 1–2 glasses of grapefruit juice per day for five days [96], or following the daily consumption of fresh grapefruit for two months [213]. In addition, rhabdomyolysis was noted in a patient taking simvastatin after the consumption of a whole grapefruit daily for two weeks [93].

Complete heart block was observed in a woman taking verapamil and consuming three to four litres of grapefruit juice for seven days per day [100]. Torsades de pointes was reported an interaction between amiodarone and grapefruit juice for a patient regularly consuming 1 to 1.5 L of juice per day [103]. Nephrotoxicity was observed in a posttransplant patient taking tacrolimus, who had ingested 1.5 kg of grapefruit-containing marmalade during the previous week [111]. Haematological toxicity was documented in a patient who consumed 250 mL of grapefruit juice daily [108]. Furthermore, an adverse drug interaction was reported in a patient taking methadone who had ingested 500 mL of grapefruit juice daily for three consecutive days prior to the onset of symptoms [109].

Coagulation disturbances were noted in a patient on warfarin therapy who consumed 680 g of cranberry juice daily [60], and in another who ingested 1420 mL of cranberry juice cocktail per day for two consecutive days [116]. A similar phenomenon was observed in a warfarin patient who consumed 3–4 glasses of concentrated herbal tea made from *Lycium barbarum* L. [120], and another who drank 4–5 cups of chamomile tea daily [62]. Furthermore, a stroke was reported in a patient treated with acenocoumarol after the consumption of 1.5 kg of liquorice candy in the days preceding the event [164].

A patient taking olanzapine suffered an exacerbation of schizophrenia symptoms which was associated with compounds present in tobacco smoke, with the patient smoking up to 80 cigarettes daily [195]. A study of 365 patients treated with selegiline as transdermal patches (20 mg/20 cm^2^), who received a diet rich in tyramine, did not demonstrate any changes differences in blood pressure compared to those receiving placebo [214]. However, increasing the dose of selegiline (30 mg/day) resulted in symptoms indicating the inhibition of MAO type A activity. As such, it is recommended that patients receiving a higher dose of selegiline should strictly limit foods containing tyramine [215].

However, it is important to emphasise that adverse drug interactions can occur even after ingesting relatively small amounts of certain food products. For example, a coagulation disorder was reported in a patient undergoing warfarin therapy who had consumed only 20 mL of Gouqizi wine the evening before the incident [121]. Similarly, an episode of priapism was documented in a patient taking sildenafil after the ingestion of just 200 mL of pomegranate juice [59].

Two cases of myocardial infarction were found to be caused by interactions with phenelzine, a MAOI. In the first case, myocardial infarction occurred approximately three hours after the consumption of an unspecified type of cheese while on phenelzine therapy [18]. In the second case, a myocardial infarction was reported after the consumption of pizza containing parmesan cheese [210].

Hence, it appears that the occurrence of FDI and HDI is largely dependent on the concentration of the interacting substances, with a higher concentration of drugs or amount of food or herbs being associated with a greater likelihood of an interaction. However, there are exceptions, where a small amount of these substances can trigger such interactions, even when consumed on one occasion.

## 10. Concluding Remarks

Scientific studies and case reports showed that compounds found in certain fruits (e.g., grapefruit or cranberry) or herbs (e.g., *Astragalus membranous* and *Schisandra sphenanthera*) act as unequivocal inhibitors of cytochrome P450 (CYP) enzymes, while others function as inducers (e.g., *Hypericum perforatum, Liquorice (Glycyrrhiza)* and *Ginkgo biloba* L.). However, in some cases, reports on CYP interactions are contradictory—as illustrated by studies on *Matricaria chamomilla* and turmeric (*Curcuma longa* L.).

The case studies and reports given in the present article can guide clinicians, providing information on food–drug or herb–drug interactions. This article can also serve as an important educational resource for patients.

Interactions between xenobiotics and food are a little-studied branch of science. Despite the everyday ubiquity of food and drugs, the lack of ongoing research or maintenance of specialised health service centres prevents the proper training of medical personnel and patients regarding their interactions. Attempts are being made to create a controlled drug delivery system to prevent these adverse interactions [216]. Research is also being carried out on the targeted use of the FDI reaction, as was the case with the successful combination of grapefruit juice and venetoclax in a patient with acute myeloid leukaemia [217].

Due to the limited public awareness of the interactions between drugs and the compounds present in food and herbs, many instances of unsafe usage have been misinterpreted as simple cases of poisoning. Pharmacists and physicians should take the initiative to educate patients about the interactions with chemical compounds present in foods and herbs, particularly since some of them can occur even with minimal consumption of certain substances.

With the present findings in mind, anyone taking drugs, including patients, should consider the following:

(1) The quantity of products consumed with the drug plays a key role in these interactions, and people should pay attention to the amount of health-promoting food and herbs consumed.
(2) The limit the intake of fruits and fruit juices when taking drugs that require CYP activity.
(3) Avoid consuming herbs such as *H. perforatum* (a CYP inducer) or *A. membranous* (a CYP inhibitor) during treatment with drugs metabolised by CYP.
(4) Limit the consumption of foods when using MAO inhibitors.
(5) Eat a varied diet with tyramine to avoid the accumulation of a single compound.
(6) Always consult a doctor or pharmacist about potential interactions when starting a new medication.

Most importantly, patients should always consult their healthcare provider before taking any drug.

## Figures and Tables

**Table 1:** Examples of potentially significant drug interactions with fruit juices during the biotransformation stage.

Mechanism of Interaction	Fruit Juice Type	Examples of Drugs	References
CYP3A4 inhibition	Grapefruit juice	Simvastatin	[58,67,68]
	Grapefruit juice	Simvastatin	[69]
	Grapefruit juice	Artemether	[70]
	Grapefruit juice	Blonanserin	[71]
	Grapefruit juice	Dapoxetine	[72]
	Seville orange juice	Sildenafil	[73]
	Seville orange juice	Felodipine	[74]
	Pomegranate juice	Sildenafil	[59,75]
	*Punica granatum* juice	Sildenafil	[59]
	Pomelo juice	Cyclosporine	[76]
CYP2C9 inhibition	Cranberry juice	Warfarin	[60]
CYP2C9 induction	Noni juice	Phenytoin	[77]
CYP3A activation	Grape juice	Cyclosporine	[78]
CYP1A2 activation	Grape juice	Phenacetin	[79]

**Table 2:** Common drugs that interact with grapefruit, according to their pharmacological group [51,52,84,85,86,87,88,89,90,91].

Classes of Drugs with Established Interactions with Grapefruit	Examples of Drugs
Cardiovascular Drugs	
Calcium channel blockers	Felodipine, nimodipine, nitrendipine, verapamil, diltiazem, nifedipine, verapamil
Antiarrhythmic drugs	Dronedarone, amiodarone
Anticoagulants	Rivaroxaban, apixaban, edoxaban
Antiplatelet drugs	Ticagrelor
Hypolipidemic drugs	Statins: atorvastatin, simvastatin
Immunosuppressants	Cyclosporine, everolimus, tacrolimus
Antibiotics	
Macrolides	Clarithromycin, erythromycin
Antimalarial	Primaquine, halofantrine, maraviroc
Anti-parasitic	Praziquantel
Antiretrovirals—HIV therapy	Saquinivir, etravirin
Antidepressants	Clomipramine, sertraline, fluoxetine, agomelatine
Other psychiatric drugs	S-carbamazepine, buspirone,, diazepam
Drugs used in the treatment of functional disorders of the upper gastrointestinal tract	Cisapride, domperidone
Oestrogens	Oestradiol, ethinyloestradiol
Drugs used in bladder diseases	Darifenacin solifenacin fesoterodine
Drugs used in prostatic hyperplasia	Tamsulosin, doxazosin
Cytotoxic agents	Nilotinib

**Table 3:** Examples of potentially significant HDIs in humans.

CYP450 Isoforms	Name of the Herb	Examples of Drug Substrate	References
Cases of increased plasma drug concentration resulting from HDI			
CYP3A4	Herbal teas	Cyclosporin	[137]
CYP3A4	Herbal Product Sheng Mai-San	Nifedipine	[138]
CYP3A4	Herbaceous astragalus (*Astragalus membranous*)	Tacrolimus	[61]
CYP3A4	*Schisandra sphenanthera*	Tacrolimus	[139]
CYP3A4	*Schisandra sphenanthera*	Midazolam	[140]
Cases of elevated plasma drug concentrations or unchanged levels resulting from HDI			
CYP2C9 inhibition	Chamomile (*Matricaria chamomilla*)	Warfarin	[62]
CYP2C9 no effect	Chamomile (*Matricaria chamomilla*)	Warfarin	[141]
CYP3A4 inhibition	Turmeric (*Curcuma longa* L.)	Tacrolimus	[63]
CYP3A4 no effect	Turmeric, curry powder, and ginger	Tacrolimus	[64]

**Table 4:** Examples of potentially significant induction of specific CYP isoforms by herbs and PAHs increasing the metabolism of certain drugs.

Induction of a Specific CYP Isoform	Name of the Herb/Herbal Preparation	Examples of Drugs	References
Cases of decreased plasma drug concentration resulting from HDI			
CYP3A4	*Hypericum perforatum*	Cyclosporine	[162]
CYP3A4	*Hypericum perforatum*	Cyclosporine	[163]
CYP2C8, CYP2C9 and CYP2C19	*Glycyrrhiza*	Acenocoumarol	[164]
CYP2C9	Noni juice	Phenytoin	[77]
CYP3A4	*Ginkgo biloba* L.	Efavirenz	[165]
CYP3A4	*Hypericum perforatum* and *Ginkgo biloba*	Fluoxetine and buspirone	[166]
CYP1A1	Polycyclic aromatic hydrocarbons	Olanzapine	[17]
Studies on herb–drug interactions in volunteers			
CYP3A4	*Hypericum perforatum*	Cyclosporine	[167]
CYP3A4	*Ginkgo biloba* L.	Haloperidol	[168,169]
CYP3A4	*Hypericum perforatum*	Alprazolam	[170]
CYP3A4	*Hypericum perforatum*	Midazolam	[54]
CYP3A4	*Hypericum perforatum*	Methadone	[171]
CYP3A4	*Hypericum perforatum*	Oxycodone	[141]
